# Inhibition of RAF dimers: it takes two to tango

**DOI:** 10.1042/BST20200485

**Published:** 2020-12-24

**Authors:** Frazer A. Cook, Simon J. Cook

**Affiliations:** Signalling Programme, The Babraham Institute, Babraham Research Campus, Cambridge CB22 3AT, U.K.

**Keywords:** BRAF, dimerisation, kinase inhibitors, paradoxical activation, RAF, RAS

## Abstract

The RAS-regulated RAF–MEK1/2–ERK1/2 pathway promotes cell proliferation and survival and RAS and BRAF proteins are commonly mutated in cancer. This has fuelled the development of small molecule kinase inhibitors including ATP-competitive RAF inhibitors. Type I and type I½ ATP-competitive RAF inhibitors are effective in BRAF^V600E/K^-mutant cancer cells. However, in RAS-mutant cells these compounds instead promote RAS-dependent dimerisation and paradoxical activation of wild-type RAF proteins. RAF dimerisation is mediated by two key regions within each RAF protein; the RKTR motif of the αC-helix and the NtA-region of the dimer partner. Dimer formation requires the adoption of a closed, active kinase conformation which can be induced by RAS-dependent activation of RAF or by the binding of type I and I½ RAF inhibitors. Binding of type I or I½ RAF inhibitors to one dimer partner reduces the binding affinity of the other, thereby leaving a single dimer partner uninhibited and able to activate MEK. To overcome this paradox two classes of drug are currently under development; type II pan-RAF inhibitors that induce RAF dimer formation but bind both dimer partners thus allowing effective inhibition of both wild-type RAF dimer partners and monomeric active class I mutant RAF, and the recently developed “paradox breakers” which interrupt BRAF dimerisation through disruption of the αC-helix. Here we review the regulation of RAF proteins, including RAF dimers, and the progress towards effective targeting of the wild-type RAF proteins

## Introduction to the RAS–RAF–MEK1/2–ERK1/2 pathway

**Figure 1.**
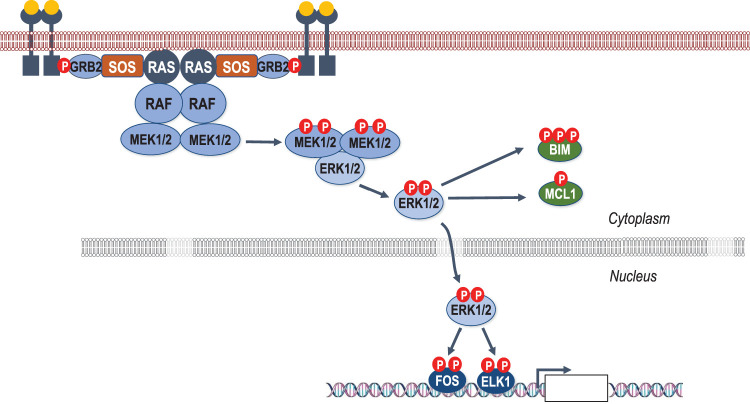
A simplified schematic representation of the RAS–RAF–MEK–ERK signaling pathway. Epidermal growth factor binding to the epidermal growth factor receptor causes it to dimerise and autophosphorylate *in trans*. This allows the binding GRB2 and SOS. This complex recruits and activates RAS, inducing RAS dimerisation and activation. Dimerised RAS recruits RAF, releasing RAF from an autoinhibited state and enabling it to recruit and phosphorylate MEK1/2. The protein components and stoichiometries at the cell membrane are poorly defined. MEK1/2 is then phosphorylated by RAF and released from the complex to bind and phosphorylate ERK1/2, activating ERK1/2 kinase activity. ERK1/2 can act in the cytoplasm to directly phosphorylate protein substrates, such as BIM to directly prevent apoptosis. ERK1/2 is also translocated to the nucleus for the activation of transcription factors such as FOS and ELK1, driving the transcription of hundreds of genes to promote cell proliferation and survival.

The RAS-regulated RAF-MEK1/2-ERK1/2 protein kinase cascade receives and amplifies signals from growth factor receptors to drive gene expression and direct cell fate, typically promoting cell survival and proliferation [1]. In the case of receptor tyrosine kinases, such the epidermal growth factor receptor (EGFR), binding of epidermal growth factor (EGF) induces receptor dimerisation and autophosphorylation, thereby activating the tyrosine kinase domain which in turn phosphorylates other residues within the intracellular C-terminal tail. These C-terminal phosphorylation sites bind GRB2 which in turn recruits Son-Of-Sevenless (SOS), a RAS-selective guanine nucleotide exchange factor which displaces GDP from RAS, allowing RAS to bind the more abundant GTP, thereby activating RAS [2]. RAS-GTP recruits RAF proteins to the plasma membrane and relieves their autoinhibited state by disrupting 14-3-3 binding to a phosphorylated serine residue, allowing Protein Phosphatase 2A access to dephosphorylate the residue and prevent 14-3-3 rebinding [3,4]. Once activated, RAF phosphorylates and activates MEK1/2, which in turn phosphorylates and activates ERK1/2, the terminal kinase in the three-tier cascade. Active ERK1/2 phosphorylate hundreds of proteins, including other kinases such RSK, but are perhaps best known for translocating into the nucleus to phosphorylate transcription factors, thereby altering the expression of hundreds of genes within the cell [5,6] (Figure 1).

The representation of RAF activated within a multimeric complex with RAS in Figure 1 is simplified since it omits key players (such as 14-3-3 proteins and the KSR1/2 pseudokinase scaffolds); in addition, the stoichiometries of the complex remain poorly defined. It is difficult to estimate with confidence the degree of signal amplification down the core cascade. Estimates of the relative protein abundance of CRAF, MEK and ERK vary from 1 : 0.7 : 9 to 1 : 3 : 6 [7], so signal amplification is clearly possible and might suggest that targeting RAF or MEK might be the most effective way to prevent ERK activation. However, key features of the cascade also limit amplification and will influence the response to inhibitors. For example, each step is facilitated by scaffold proteins which bring components together to make phosphorylation more efficient; however, these scaffolds typically engage one molecule of kinase (e.g. RAF) and substrate (e.g. MEK) at a time so this will also limit amplification. In addition, negative feedback loops driven by ERK-dependent phosphorylation of upstream components (SOS, RAF, MEK) limit pathway activation; indeed, the core cascade has the properties of a negative feedback amplifier [7] and this also allows the pathway to respond to interventions such as RAF, MEK or ERK inhibitors. These control mechanisms also allow fine-tuning of ERK1/2 activation which is critical since the magnitude and duration of ERK1/2 activation can elicit quite different biological responses. This is strikingly illustrated by a recent report in which mass spectrometry was used to provide absolute quantification of total and phosphorylated (activated) ERK1 and ERK2 [8]; this revealed that just 3-5% of ERK1/2 is active in tumour cells with mutant BRAF^V600E^. MEK inhibitor-resistant versions of the same cells exhibited the same level of ERK1/2 activation despite extensive amplification of BRAF^V600E^. Indeed, resistant cells adapt to become to addicted to the MEK inhibitor to maintain this level of ERK1/2 activation; withdrawal of the drug leads to activation of up to 20–30% of the ERK1/2 and results in sustained cell cycle arrest and cellular senescence or cell death. This study demonstrates that the fraction of ERK1/2 that is activated must be kept within a very narrow range to support cell proliferation and also reveals substantial spare capacity in the pathway which is likely kept in check by feedback loops and the action of DUSP family of ERK1/2 Phosphatases [8]

The *RAS* genes are the most commonly mutated oncogenes in cancer, with ∼16% of cancers containing a mutant RAS protein [9]. Mutations are most frequently located at amino acid residues G12, G13 and Q61. These residues all have key roles in the GTPase function of RAS; mutation of these residues reduces GTPase activity, in large part by making the protein refractory to GTPase-activating proteins (GAPs); consequently these mutant RAS proteins persist in the active GTP-bound conformation [10] driving persistent activation of effector pathways including ERK1/2 signalling. KRAS mutations are common in lung adenoma, pancreatic ductal cancer and colorectal cancer whilst NRAS mutations are found in melanoma [9]. *BRAF* mutations occur in almost all (>97%) hairy cell leukaemia [11], ∼50% of melanoma [12] and conventional papillary thyroid cancers [13], and up to 7% of lung adenomas [14]. MEK mutations are very rare and can be either RAF-independent, RAF-regulated or RAF-dependent [15]. Dysregulated ERK1/2 signalling contributes to several of the hallmarks of cancer [16,17], such as sustained proliferative signalling [18], angiogenesis [19], resistance to cell death [20,21] and invasive behaviour leading to metastasis [22]. Many of these hallmarks reflect deregulated activity of ERK1/2-dependent transcription factors such as ETS, FOS/AP-1 and MYC.

The frequent mutational activation of BRAF in melanoma [23] led to the development of type I small molecule inhibitors to inhibit the constitutive activity of the most common RAF variant in cancer, BRAF^V600E^, which is active as a monomer in contrast with wild-type RAF which signals as a dimer. These type I compounds are successful in the treatment of BRAF^V600E^-mutant melanoma, but are limited by their failure to inhibit wild-type RAF; rather, they induce dimerisation of wild RAF proteins by stabilising the dimer interface, leading to paradoxical activation of MEK1/2 and thence ERK1/2 through the uninhibited dimer partner [24]. This has led to interest in RAF dimers and drug-induced paradoxical activation of the RAS–RAF–MEK1/2–ERK1/2 pathway.

## Mutation of the RAF proteins in cancer

There are three major classes of BRAF mutations. Class 1 BRAF mutants are predominantly V600 variants, with BRAF^V600E^ the most common; they do not require dimerisation to activate MEK1/2 and signal constitutively as monomers independently of RAS by mimicking phosphorylation of the active loop [25]. Class 2 BRAF mutants such as G469V are inactive as monomers but dimerise and activate MEK1/2 independently of RAS interaction. Class 3 BRAF mutants such as G466V have inhibited or ablated kinase activity. These RAF mutants still dimerise with other RAF proteins in a RAS-dependent manner but their inactive kinase domain means they cannot auto-phosphorylate inactivating residues and thus are held in an active conformation for longer; this extends the activation of RAF dimer partners and the transactivation of MEK1/2 phosphorylation in their dimer partner [26]. Class 2 and 3 BRAF mutants are frequently located in the P-loop in residues responsible for engaging the phosphate groups of ATP. Some BRAF mutations do not fit into these three categories and remain unclassified [27]. ARAF and CRAF mutations are far rarer in cancer and seem to behave like class 3 BRAF variants. ARAF^S214C^ [28,29] and CRAF^S259A^ [30] have each been shown to have a reduced kinase activity and therefore are likely to be oncogenic by transactivating their dimer partner. Each of these mutations result in increased active MEK1/2-ERK1/2, thereby promoting cell proliferation and survival.

## The discovery of RAF dimerisation and its role in RAS-dependent ERK activation

RAF dimerisation is characterised by a side-to-side interaction of two RAF proteins to form a catalytically active protein dimer. Wild-type RAF protein dimerisation is driven by active RAS, activating RAF phosphorylations, 14-3-3 protein binding and binding of KSR1/2, and although mutation of key residues within the RAF dimer interface can disrupt the formation of RAF dimers [31] an intact RAF dimer interface is not sufficient to drive RAF dimerisation and kinase activity independently of these other proteins [32].

Evidence of RAS activating ERK1/2 was first provided by Sally Leevers and Chris Marshall [33]. Subsequent identification of MEK1 as an ERK1/2-activating kinase, identification of RAF proteins as MEK activators and identification of RAF proteins as direct targets of RAS-GTP has been reviewed extensively [34,35]. The first evidence for RAF dimerisation driving MEK1/2 activation was obtained using a chimeric CRAF-GyraseB fusion protein, which allowed coumermycin-dependent dimerisation [36]. This study showed that forced dimerisation of otherwise wild-type RAF proteins activated MEK1/2. A similar study employed a CRAF-FKBP (FK506 binding protein) fusion and treatment with FK1012A to induce dimerisation [37]; induction of dimers activated CRAF, although to a lower degree than RAS-mediated dimerisation. The role of RAS in the formation of BRAF:CRAF heterodimers was elucidated by the co-immunoprecipitation of CRAF and BRAF in the presence of KRAS^G12V^, an activated oncogenic KRAS variant [38]. This was the first study to show RAF dimerisation without using artificial dimerisation constructs and also showed that MEK1/2 activation by BRAF:CRAF heterodimers was caused by the RAS-mediated recruitment of RAF dimers to the plasma membrane, as a KRAS^G12V^ variant (aa 1–166) lacking C-terminal membrane targeting residues failed to increase MEK1/2 phosphorylation despite co-immunoprecipitating with, and increasing the formation of, BRAF:CRAF heterodimers.

Studies have shown that RAS binds to conserved region 1 (CR1) [39–41] whilst the coordination of a zinc ion to the plasma membrane by the cysteine-rich region of CR1 further stabilises the RAS–RAF interaction [42]. Evidence also suggests that the CRAF kinase domain interacts with the membrane at phosphatidic acid-rich sites as the RKTR motif of the αC-helix, the NtA (N-terminal acidic) region and the activation segment form an interface that is similar in structure to the membrane-binding domains of other proteins [43]. RAF recruitment to the plasma membrane by RAS is important for relieving the binding of 14-3-3 proteins, suppressors of RAF activation. RAF proteins contain a conserved C-terminal 14-3-3 binding site (BRAF Ser^728^), and a second conserved serine residue within CR2 (BRAF Ser^364^) providing a bivalent 14-3-3 binding site. This inhibition is relieved by interactions with prohibitin, a plasma membrane-bound protein, which is thought to displace the 14-3-3 protein from CR2 allowing access of Protein Phosphatase 2A [3] to dephosphorylate the CR2 serine residue. Recent work has shown that the SHOC2 complex is able to dephosphorylate the conserved serine residue of CR2 and prevent re-binding of 14-3-3, sustaining dimerisation and RAF activation [44].

The role of 14-3-3 binding proteins in RAF dimerisation and activation has recently been made clearer through a study aimed at identifying the role of ATP binding in RAF dimerisation [45]. Binding of the ATP homologue ACP reduced the dimerisation affinity of BRAF:MEK tetrameric complexes (2 BRAF, 2 MEK proteins), which was overcome by the addition of 14-3-3 protein. Whilst previous studies have shown that 14-3-3 protein binding is important for MEK1/2 activation and that the mutation of the C-terminal 14-3-3 binding site can reduce pathway activation [46], this paper appears to be the first to identify the role of 14-3-3 in stabilising the BRAF:MEK complex and preventing a twist in the N-lobe of RAF protein. Recent work has also suggested that 14-3-3 maintains active BRAF dimers within the cell by binding to and bridging the C-terminal 14-3-3 binding sites of each monomer [47], and that 14-3-3 binding maintains a single RAF monomer in an active conformation whilst rendering the other RAF monomer in an inactive conformation, potentially offering some insight into the activating effects of kinase-dead class III RAF mutants [48]. 14-3-3 protein interactions are currently under investigation to confirm whether this is a druggable interaction to prevent RAF dimerisation.

## The structure of RAF proteins

**Figure 2.**
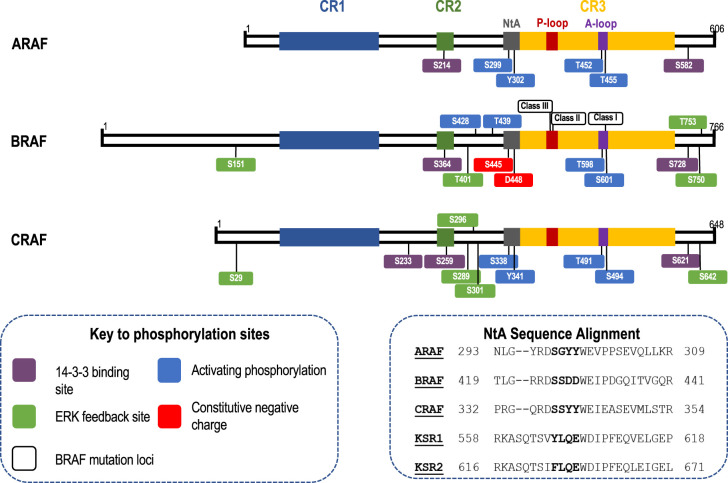
Comparison of RAF structures and their phosphorylation sites. The three RAF proteins, ARAF, BRAF and CRAF each share three conserved regions; CR1, CR2 and CR3. CR1 is essential for binding to RAS at the plasma membrane, CR2 contains a binding site for 14-3-3 protein, and CR3 is the kinase domain and includes the phosphorylation loop (P-loop), activation loop (A-loop) and is responsible for RAF dimerisation and phosphorylation of MEK1/2. The glycine rich P-loop is important for binding ATP. Phosphorylation of the threonine and serine residues within the activation loop is essential for the kinase function of each RAF protein. Adjacent to CR3 is the N-terminal acidic region (NtA). Two residues of the NtA need to be phosphorylated for ARAF (S299, Y301, Y302) and CRAF (S338, S339, Y340, Y341) to dimerise, whereas BRAF is constitutively phosphorylated at the serine residues (S445, S446) and has an aspartic acid residue instead of a tyrosine (D447, D448) to impart a constitutive negative charge. This region interacts with the aC-helix to form the RAF dimerisation interface. The C-terminus of each RAF protein contains a second binding site for 14-3-3 proteins. BRAF and CRAF each contain several ERK phosphorylation sites that form part of negative loops to reduce RAF activity. Several of these feedback sites are conserved between RAF species. Loci for BRAF mutations are also shown, with class I BRAF mutations occurring within the activation loop at V600, class II mutations frequently occurring at G469 in the P-loop, and class III mutations frequently occurring at G466 in the P-loop. Finally, a sequence alignment of the NtA sequence of the ARAF, BRAF, CRAF, KSR1 and KSR2 is shown. The serine and tyrosine residues in ARAF and CRAF are able to be phosphorylated, with the serine residues of BRAF being constitutively phosphorylated. The NtA region in KSR1 contains a tyrosine residue which can be phosphorylated and may play a role in activating BRAF.

The three RAF proteins are encoded by distinct genes; *ARAF, BRAF* and *CRAF*. RAF proteins form homodimers (e.g. BRAF:BRAF) and heterodimers (e.g. BRAF:CRAF); mutant RAS proteins preferentially promote BRAF:CRAF heterodimers which are also the most effective dimer pair for activating MEK1/2 [49]. ARAF, BRAF and CRAF each share three ‘conserved regions’ (CRs) (Figure 2). CR1 consists of a RAS binding domain that binds RAS-GTP [50], and a zinc-binding cysteine rich domain which further stabilises RAF interactions with RAS at the plasma membrane [51]. CR2 contains a conserved serine residue which, together with a conserved N-terminal site binds 14-3-3 proteins when phosphorylated [3,52]. CR3 consists of the catalytic kinase domain, and is responsible for binding and phosphorylating MEK1 or MEK2 and includes the catalytic DFG motif and the regulatory αC-helix domain. Phosphorylation of the RAF activation loop drives a conformational change in the protein, altering the RAF structure from DFG-out to DFG-in and αC-helix-out to αC-helix-in conformation, inducing an active protein conformation [53]. Adjacent to CR3 is the NtA region containing a serine residue in each RAF species and a tyrosine residue in ARAF and CRAF. In BRAF the tyrosine residue is replaced with an aspartic acid residue and the serine residue is constitutively phosphorylated, imparting a constitutive negative charge to the region. The NtA motif enables RAF species to allosterically activate their dimer partner [54]. ARAF and CRAF activation therefore requires the phosphorylation of both the tyrosine and serine residues at this region (Figure 2). This also means that BRAF is able to be activated by a single point mutation due to the constitutive priming for dimerisation imparted by these constitutive negative charges.

The RAF dimer interface is also partially conserved in the Kinase Suppressors of RAS (KSR) protein family. These are pseudokinases sharing significant structural homology with RAF proteins. KSR1 and KSR2 are thought to play a role in the activation and dimerisation of BRAF:MEK complexes, potentially offering an alternative manner of activation to the conventional RAS-driven activation of RAF. KSR1 contains a tyrosine within a conserved motif from the NtA region (Figure 2), which could explain why KSR1 is able to transactivate BRAF unlike KSR2 [55]. A recent study has also shown the potential of targeting KSR1/2:MEK1/2 complexes, offering a potential therapeutic approach for targeting aberrant ERK1/2 signalling [56,57].

RAF kinase activation is achieved through a phosphorylation-dependent realignment of two spines along the length of the protein, crossing both N- and C-terminal lobes [53]. The first of these, the regulatory spine, contains both the αC-helix and DFG sequence of the activation loop. The alignment of four hydrophobic residues stabilises a closed, active conformation of the protein, with the phenylalanine of DFG and an aliphatic residue of the αC-helix aligning alongside the HRD motif of the catalytic loop and a residue from the β4-strand of the protein. The regulatory spine is completed upon the adoption of a DFG-in, αC-helix-in conformation. Subsequently, the catalytic spine, formed of 8 hydrophobic residues, is aligned when RAF binds ATP [58] and is necessary for hydrolysis of ATP to ADP and the phosphorylation of MEK1/2.

## The RAF dimer interface

RAF proteins dimerise through side-to-side interactions of interfaces formed by the CR3 kinase domain [59]. These interfaces are dynamic in the inactive DFG-out, αC-helix-out, open conformation and as a result are not stable enough for dimer formation. Alignment of the regulatory spine and adoption of the αC-helix-in, DFG-in closed, active conformation stabilises this interface, allowing interactions between two RAF monomers to form an active dimer [60]. The RKTR motif within the αC-helix (R506, K507, T508 and R509 in BRAF) is important for modulating RAF dimerisation [31]. Substitution of these residues with alanine, especially BRAF^R506A^, reduced BRAF dimerisation [61]; similarly, mutation of either Arg residues or the Lys residue within ARAF or CRAF reduced dimer formation. The RKTR motif is correctly positioned for dimerisation after the alignment of the regulatory spine and adoption of an αC-helix-in conformation so RAF dimerisation is intrinsically linked to the adoption of an αC-helix-in conformation and RAF activation [59]. The positive charge of the RKTR motif interacts with the phosphorylated NtA region of the partner RAF protein after phosphorylation of the Tyr and Ser residue in ARAF [62] and CRAF [63], whilst BRAF contains an Asp in place of the Tyr and is constitutively phosphorylated at the Ser, forming a salt bridge that stabilises the dimer. The constitutive negative charge within the BRAF NtA region leaves the protein ‘primed’ for dimerisation, and able to be activated by single site phosphorylation or single point mutations [64]; this may explain why BRAF mutations are far more common in cancer than ARAF or CRAF mutations. The NtA region residues are correctly positioned by a conserved tryptophan residue, W450 in BRAF; mutation to Ala decreases the kinase activity of wild-type BRAF, but not BRAF^V600E^; thus this residue is important for the activation of BRAF dimers but not for activity of the common oncogenic BRAF mutants that signal as monomers [54].

## ERK1/2-mediated feedback inhibition regulates RAF dimerisation

**Figure 3.**
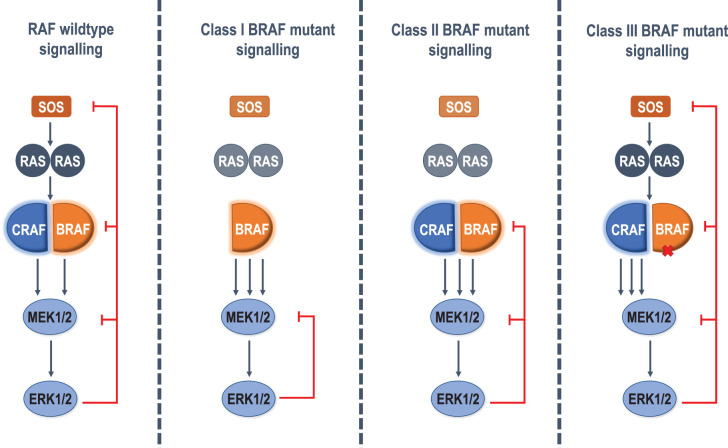
Direct ERK1/2 feedback inhibition of RAF mutants. ERK1/2-mediated feedback inhibition down-regulates signalling by ERK1/2-catalysed phosphorylation of SOS, RAF and MEK1/2 to prevent hyperstimulation of ERK signalling. SOS phosphorylation reduces RAS activation. Phosphorylation of RAF prevents dimerisation and therefore kinase signalling. Finally, feedback phosphorylation of MEK1 prevents its activation. Class I BRAF mutants signal as monomers regardless of RAS activity, and are unaffected by ERK1/2 feedback inhibition through SOS and the disruption of RAF dimer formation. Class II BRAF mutants signal constitutively as dimers independently of RAS activity so they are susceptible to feedback disruption of RAF dimers and MEK1/2 activation but are not susceptible to SOS phosphorylation. Finally class III RAF mutants are still reliant on RAS for dimerisation and activity, and therefore are susceptible to all direct feedback inhibition mechanisms of ERK1/2.

Whilst deregulated ERK1/2 signalling can be oncogenic [34] it can also promote cell death or senescence [65–67] depending on the magnitude of ERK1/2 activation; as a result the ERK1/2 signalling pathway is tightly regulated by feedback controls [68]. Some of these feedback controls are indirect; for example ERK1/2 activation drives the *de novo* expression of dual-specificity phosphatases (DUSPs) such as DUSP5 and DUSP6 that dephosphorylate the critical T-E-Y motif contained within the activation loop of ERK1/2, inactivating ERK1/2 [69]. However, the ERK1/2 signalling pathway is also regulated by direct negative feedback phosphorylation of key components by active ERK1/2 (Figure 3). SOS1 and SOS2 are phosphorylated on Ser residues after treating cells with EGF [70] either by ERK1/2 directly or indirectly through other kinases such as RSKs [71,72], which are phosphorylated and activated by ERK1/2 [73]. Phosphorylation of SOS1 by ERK1/2 reduces the association of SOS1 and Grb2, thereby preventing RAS activation [70]. MEK1 is phosphorylated by ERK1/2 at T292, preventing an activating phosphorylation of S298 by p21 Activated Kinases (PAKs) and therefore phosphorylation of ERK1/2 [74].

Critically, the RAF proteins are also direct targets of ERK1/2-mediated feedback inhibition and this reduces their dimerisation (Figures 2 and 3). RAF feedback phosphorylation in response to ERK1/2 activation was first identified in 1994 [75]. Dual phosphorylation of a specific SPKTP motif within BRAF and CRAF was shown to down-regulate ERK1/2 activation, [76], with phosphorylation of BRAF residues S750 and T753 impairing BRAF:CRAF dimerisation and reducing ERK1/2 activation [77]. Additionally, phosphorylation of S289, S296 and S301 of CRAF by ERK1/2 has also been shown to inactivate RAF signalling [78]. ERK1/2-dependent BRAF phosphorylation reduces heterodimer formation due to reduced affinity for RAS (phosphorylation at S151) and direct disruption of BRAF:CRAF heterodimers (pT401, pS750 and pT753) [79]

As ERK1/2 feedback phosphorylation impairs RAF dimerisation it is effective at blocking wild-type RAF activity. However, since class 1 BRAF mutants (e.g. BRAF^V600E^) signal constitutively as monomers, independent of RAS, they are not susceptible to either the ERK1/2-mediated feedback phosphorylation of SOS and inhibition of RAS activation or the inhibition of RAF dimerisation imparted by direct phosphorylation of RAF proteins by ERK1/2. Class 2 RAF variants dimerise independently of RAS so are unaffected by ERK1/2-mediated phosphorylation of SOS and RAS inactivation. However, class 2 RAF mutants are subject to ERK1/2-mediated inhibition via direct phosphorylation and inhibition of dimerisation. Thus liberation from ERK1/2-mediated feedback phosphorylation may contribute to the oncogenic properties of mutant RAF proteins and their response to inhibitors (Figure 3).

## RAF inhibitors (RAFi) and paradoxical activation

The RAS–RAF–MEK–ERK cascade amplifies signal from RAF to MEK to ERK [7], meaning that blocking signalling at the RAS or RAF level may lead to significantly greater reduction in ERK1/2 activation. BRAF is commonly mutated independently of RAS activity and signal amplification mainly occurs between RAF, MEK and ERK, making RAF a desirable target. In addition to RAS-independent activation of RAF signalling through class I and II mutants, RAS is also a difficult target due to a paucity of binding pockets [80] and high affinity for GTP [81]. However, successful targeting of the KRAS^G12C^ mutant has now provided a proof-of-concept that potent selective RAS inhibitors can be found [82].

The first drug to be approved with activity against the ERK1/2 pathway was the pan-RAF inhibitor Sorafenib [83] but efforts to inhibit RAF were galvanised by the discovery of BRAF^V600E^ mutations in melanoma [23,84]. Three BRAF inhibitors - vemurafenib, dabrafenib and encorafenib — are now approved for treatment of BRAF^V600E/K^ mutant melanoma. These bind within the ATP-binding pocket of the protein competing with ATP and preventing phosphorylation of MEK1/2. However one undesirable effect of these drugs is the paradoxical activation of ERK1/2 in cells with wild-type RAF. RAFi targeting class I BRAF mutants recognise and bind the active form of the protein, targeting the DFG-in and αC-helix-in motifs of the protein. Whilst these inhibitors are effective at targeting the mutant BRAF protein, they can also lock wild-type RAF protein dimers into active conformations and drive the paradoxical activation of MEK1/2 and thence ERK1/2. The enhanced ERK1/2 activation in RAF-wild-type cells means that ∼20% of patients treated with vemurafenib or dabrafenib develop non-metastatic skin tumours [85,86], pre-malignant colonic adenomas and gastric polyps after RAF inhibitor treatment [87].

**Figure 4.**
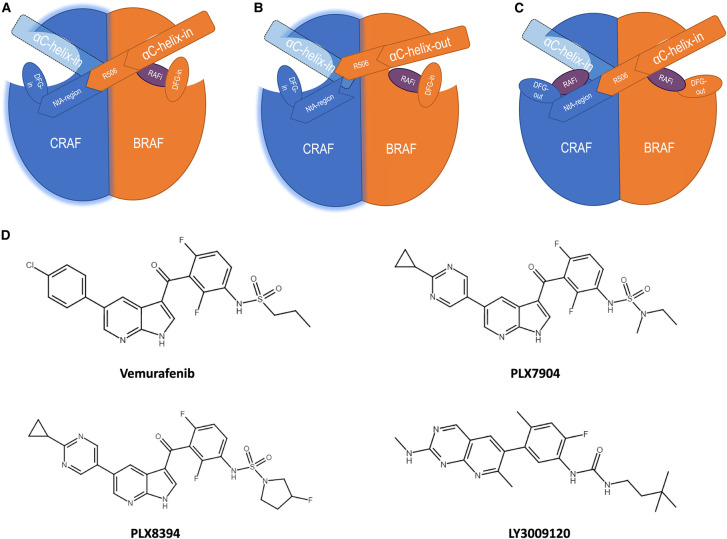
RAF conformations bound by type I, type I½ and type II RAF inhibitors. Type I, I½ and type II RAF inhibitors (RAFi) each bind to different conformations of RAF. (**A**) Type I RAFi bind to a closed, active conformation of RAF with DFG-in, αC-helix-in. Whilst this is effective for the inhibition of class I BRAF mutants, the closed conformation is conducive to dimerisation of wild-type RAF. Because class I RAFi are specific for BRAF this means the CRAF dimer partner is uninhibited by class I RAFi and thus is transactivated. (**B**) Type I½ RAFi bind to a DFG-in, αC-helix-out conformation. These inhibitors lower the binding affinity for the dimer partner because of the partial disruption of the dimerisation interface, as the binding of a second inhibitor would require breaking the dimer which is not energetically favoured at low concentrations of inhibitor. High concentrations of type I½ RAFi are able to inhibit both dimer partners and prevent pathway activation. (**C**) Type II RAFi bind to a DFG-out, αC-helix-in conformation. As type II inhibitors are pan-RAF rather than BRAF-selective they are able to inhibit pathway signalling despite promoting RAF dimerisation. (**D**) The structures of Vemurafenib, PLX7904, PLX8394 and LY3009120. Vemurafenib was used as a skeleton for the development of PLX7904, which was then optimised into PLX8394 to selectively target BRAF^V600E/K^ [92]. Whilst LY3009120 shares some structural similarities with vemurafenib, PLX7904 and PLX8394, LY3009120 does not contain a sulphonamide group and is a pan-RAF inhibitor targeting both BRAF and CRAF.

Paradoxical activation is driven by type I or type I½ RAFi. Each bind in an ATP-competitive manner but recognise different conformations of RAF protein (Figure 4). Type I inhibitors such as GDC-0879 bind to the active DFG-in, αC-helix-in conformations of RAF; this places RAF in a thermodynamically stable, closed conformation and aligns the regulatory spine. Type I½ RAFi also stabilise another regulatory spine, the ‘regulatory spine or R’ spine’, which is assembled upon adopting a closed, active conformation and can be disrupted by kinase inhibitors [88]. This promotes dimerisation with other RAF proteins and paradoxical activation of MEK1/2 and ERK1/2 at low concentrations of RAFi. Type I½ inhibitors such as dabrafenib and vemurafenib bind to the DFG-in, αC-helix-out conformation. Inhibitors which stabilise the αC-helix-out conformation partially disrupt RAF dimers after binding to a single RAF protein by displacing a single αC-helix:NtA-region salt bridge that is important for stabilising RAF dimers; however they do not disrupt the second αC-helix:NtA-region salt bridge as this would require a second inhibitor to bind and break the dimer which is not energetically favoured [31,88]. This allows transactivation of the dimer partner and an increase in ERK1/2 signalling. Type I and I½ inhibitors each effectively target BRAF^V600E^ but paradoxically activate RAF at low concentrations in cells with wild-type RAF [89,90]; however increasing inhibitor concentration to occupy both dimer partners overcomes paradoxical activation resulting in a bell-shaped dose-response curve for ERK1/2 activation [91].

Binding of Type I and I½ RAFi to one protomer of the dimer decreases the affinity for binding the dimer partner and the occupancy time of the site. Type I and I½ RAF inhibitors are therefore effective in the treatment of class I BRAF V600 mutations where MEK activation is stimulated by monomeric BRAF^V600E^ and any effect on dimer formation is unimportant. However, in such cases mechanisms of acquired resistance to Type I and I½ RAFi include deletions which mimic class II RAF mutations that are able to dimerise independently of RAS activation [93]. This re-enables RAF signalling through paradoxical activation and transactivation of the dimer partner. Other acquired resistance mutations include the BRAF^T529M/I^ gatekeeper mutation, which makes cells resistant to RAFi by preventing inhibitor binding in the ATP pocket whilst still allowing for ATP binding and signalling [94]. Class I BRAF mutant melanomas also acquire resistance to RAFi via increases in RTK and NRAS expression, which increasing the proportion of active RAF [95]. Whilst the specific structures of RAFi-bound RAF and RAF dimers are beyond the scope of this review, several extensive reviews on this topic are available [92,96].

Resistance to RAFi develops relatively quickly, with median progression-free survival for dabrafenib monotherapies being ∼9 months [97]. Presently, MEK inhibitors (MEKi) are used in tandem with RAFi treatments to both increase the median progression-free survival of patients and to prevent the emergence of paradox-induced tumours. Co-treatment with a MEKi reduces the incidence of paradox-induced tumours from 19% to 2–7% [98] but MEKi can have some severe side effects and acquired resistance to the combination treatment still occurs at ∼11 months [97]. New RAFi have therefore been sought to inhibit BRAF^V600E^ signalling without causing paradoxical activation wild-type RAF and to overcome rapid resistance to RAFi by targeting both RAF monomers and dimers.

## Breaking the paradox

Two different strategies are currently under investigation for the inhibition of mutant RAF signalling, whilst mitigating paradoxical activation — type II pan-RAF inhibitors and paradox breakers. Type II RAFi target DFG-out and αC-helix-in conformations of RAF proteins (Figure 4). This is important as they are able to target both active RAF dimers and active RAF monomers at similar potencies and therefore inhibit ERK signalling in cells with either active RAF monomers or dimers. By locking RAF protein in an αC-helix-in conformation these drugs strongly induce dimerisation of RAF protein; however, as BRAF and CRAF are targeted at similar potencies transactivation of the dimer partner is mitigated. Examples of type II RAF inhibitors include AZ628, belvarafenib, CCT196969, CCT241161, LY3009120 and TAK-580 (MLN2480). Whilst these drugs have been effective *in vitro* [99,100], their uses *in vivo* have been more limited [101] due to their lack of selectivity for mutant BRAF, which contrasts with the first-generation inhibitors such as vemurafenib and dabrafenib. This could potentially lead to greater toxicity within non-cancer cells and greater disruption of wild-type RAF signalling, although the example of sorafenib shows that some pan-RAF inhibitors can be well tolerated [102,103]. These drugs elicit partial responses in NRAS-mutant melanoma, and in some BRAF-mutant cancers [100]. Type II RAFi could potentially become part of standard care as a way to limit ERK1/2 signalling resulting from both mutant RAF proteins and RAS-driven, wild-type RAF proteins, as these are pan-RAF specific and so will not induce paradoxical activation. They may obviate some common mechanisms of acquired resistance to BRAF^V600E/K^-selective inhibitors; for example, type II inhibitors should mitigate the switch from BRAF^V600E^ to ARAF or CRAF that is observed in melanoma. However, it would need to be shown that the drugs are able to work within a window which is deleterious to cancer cells but does not strongly affect wild-type ERK1/2 signalling. Studies utilising these drugs as part of a co-treatment with MEK inhibitors are ongoing to see if this will improve the response rate within patients.

Paradox breakers are a class of BRAF inhibitors recently developed by Plexxikon [104]. Compounds were developed utilising vemurafenib's structure to selectively bind BRAF and various terminal sulphonamide and sulfamide modifications. Candidates were screened in cells for inhibition of ERK1/2 in BRAF^V600E^ cells without driving paradoxical activation of ERK1/2 in RAS-mutant cell lines. PLX7904 was identified from these screens, with a more optimised structure, PLX8394, developed shortly thereafter. These drugs bind strongly to Leu505, immediately adjacent to the RKTR motif of the αC-helix. This disrupts the RAF dimer interface, preventing the induction of BRAF:CRAF heterodimers normally observed in RAS-mutant cells treated with RAF inhibitors, without lowering the binding affinity of the drug for the dimer partner. In one study, cell lines were generated from a patient with concurrent BRAF^V600E^-mutant melanoma and KRAS-mutant colorectal cancer, with paradox breakers inhibiting the BRAF^V600E^-mutant cells without paradoxically enhancing the growth of the KRAS-mutant cells [105]. Paradox breakers are more specific for mutant BRAF, specifically BRAF V600 mutants, than WT-RAF proteins. This means that they would not be appropriate for treating RAS-mutant cancers, however they could potentially be a future replacement for type I½ RAFi for the treatment of BRAF mutant cancers such as most melanoma and hairy-cell leukaemia without paradoxically inducing tumour formation. Additionally, efficacy of these paradox breakers was maintained in cancers with dimerisation-mediated resistance to vemurafenib [104] suggesting that paradox breakers could be used after resistance to type I½ RAFi has developed.

PLX8394 has also shown some efficacy against BRAF protein fusions [106]. BRAF fusions contain the catalytically active CR3 kinase domain but lack the regulatory CR1 and CR2 domains [107]. In some melanoma cell lines driven by BRAF fusions with a 5′ partner that contains a dimerisation domain, PLX8394 has been shown to paradoxically activate RAF signalling [108]. One possible cause for the lack of efficacy of PLX8394 in some BRAF fusion cancers could be that BRAF:BRAF homodimers each contain the constitutively primed NtA region meaning that, even though one partner may be inhibited by PLX8394, transactivation of the dimer partner may still be possible if this interaction is stabilised by dimerisation of the 5′ fusion partner. BRAF fusions could potentially be driving the acquired resistance to PLX8394 noted in some pre-clinical trials, in addition to increased RTK expression and mTOR activity potentially compensating for the reduction in p-ERK1/2 [109]. The paradox breakers are currently under a stage I/IIa trial (NCT02428712).

## Conclusions

The RAS–RAF–MEK–ERK signalling pathway is commonly dysregulated in cancers driving inappropriate cancer cell proliferation and survival. Mutant RAF proteins drive ERK1/2 activation by signalling as monomers, dimers, or by lowering the kinase activity of one protein monomer and transactivating dimer partners. RAF has easily accessible binding pockets for small molecule inhibitors, and RAFi have been approved for the treatment of BRAF^V600E/K^-mutant melanoma. However, the paradoxical transactivation of WT RAF and resultant ERK1/2 activation by RAFi and acquired dimer-mediated resistance mechanisms has limited the further use of RAFi.

RAF dimerisation is increased by RAS activation and reduced by ERK1/2-mediated negative feedback phosphorylation; these promote or impair the adoption of a closed, active conformation of RAF protein and alignment of the regulatory spine. RAF forms dimers through the interaction of a key RKTR motif from this regulatory spine and the NtA-region of a RAF dimer partner, stabilised at the inner cell membrane by active RAS. While this would logically mean that to inhibit the formation of RAF dimers and limit RAF activity this αC-helix would need to be disrupted, this instead drives the formation of active dimers and paradoxical activation of the pathway when only a single monomer is inhibited at lower concentrations of inhibitor. This has been mitigated by use of MEKi as co-treatments, however median progression-free survival is still under a year.

Class II RAF inhibitors are currently undergoing clinical trials aimed at treating RAF-mutant cancers without inducing paradoxical activation. These are able to effectively target both active RAF monomers and RAF dimers so that no paradoxical pathway activation is induced. Whilst their pan-RAF nature would make them suitable for class I, II, and III RAF mutant cancers, deeper understanding of how the effectiveness of these compounds is limited *in vivo* when compared with *in vitro* is key to their future use. The paradox breakers, PLX7904 and PLX8394, are effective at treating both BRAF-mutant and BRAF-inhibitor-resistant cancers without inducing RAF dimerisation and paradoxical activation of the ERK1/2 pathway. The first results from clinical trials with these paradox breakers are eagerly awaited. Finally the discovery of RAF dimers, their properties and paradoxical RAF activation has highlighted the continuing importance of understanding the basic biology and biochemistry of the RAS–RAF–MEK1/2–ERK1/2 pathway.

## Perspectives

- **Importance of the field:** Whilst mutant BRAF^V600E^ is readily targeted by RAF inhibitors in melanoma, paradoxical activation of ERK1/2 signalling in cells with wild-type RAF and dimer-mediated acquired resistance to RAF inhibitors has limited their wider use. The development of RAFi that can target both monomeric and dimeric active RAF without driving paradoxical ERK1/2 signalling could be an effective treatment for a wide variety of cancers.
- **Current thinking:** Whilst dimerisation can now be uncoupled from activation by type II inhibitors, and dimerisation can now blocked effectively by paradox breakers, these compounds are not yet clinically approved for treating patients.
- **Future directions:** Identifying any toxicity concerns with type II inhibitors in the clinic could allow for the effective treatment of RAF-mutant cancers. Investigating combinations of these class II inhibitors with MEK inhibitors in the clinic is currently planned, and could potentially enhance pathway inhibition. The paradox breakers PLX8394 and PLX7904 are currently undergoing clinical trials and could prove more effective drugs for the treatment of class I BRAF-mutant cancers.

## Abbreviations

CR1: conserved region 1
EGF: epidermal growth factor
SOS: Son-Of-Sevenless
